# Claudin 1 in Breast Cancer: New Insights

**DOI:** 10.3390/jcm4121952

**Published:** 2015-11-27

**Authors:** Bowen Zhou, Amanda Moodie, Anne A. A. Blanchard, Etienne Leygue, Yvonne Myal

**Affiliations:** 1 Department of Pathology, Faculty of Health Sciences, University of Manitoba, Winnipeg, MB R3E3P5, Canada; zhoub34@myumanitoba.ca (B.Z.); moodie-a@webmail.uwinnipeg.ca (A.M.); anne.blanchard@umanitoba.ca (A.A.A.B.); 2 Department of Physiology and Pathophysiology, Faculty of Health Sciences, University of Manitoba, Winnipeg, MB R3E0J9, Canada; 3 Department of Biochemistry and Human Genetics, University of Manitoba, Winnipeg, MB R3E0J9, Canada; Etienne.leygue@umanitoba.ca

**Keywords:** claudin 1, breast cancer, EMT, “high claudin”, collective migration, interacting partners

## Abstract

Claudin 1 is a small transmembrane protein responsible for maintaining the barrier function that exists between epithelial cells. A tight junction protein that regulates the paracellular transport of small ions across adjacent cells, claudin 1 maintains cellular polarity and plays a major role in cell-cell communication and epithelial cell homeostasis. Long considered to be a putative tumor suppressor in human breast cancer, new studies suggest a role much more complex. While most invasive breast cancers exhibit a down regulation or absence of claudin 1, some aggressive subtypes that exhibit high claudin 1 levels have now been described. Furthermore, a causal role for claudin 1 in breast cancer progression has recently been demonstrated in some breast cancer cell lines. In this review we highlight new insights into the role of claudin 1 in breast cancer, including its involvement in collective migration and epithelial mesenchymal transition (EMT).

## 1. Breast Cancer

Breast cancer is the most common cancer in women and the second leading cause of cancer death in women worldwide [1]. A difficulty in treating this disease is attributed to the heterogeneous nature of breast cancer, which can be classified into a growing number of molecular subtypes (10 to date), many poorly characterized [2]. Of these, the most characterized are the luminal A, luminal B, human epidermal growth factor receptor 2 (HER2) over expressing, the basal-like, and more recently, the claudin-low subtype [3]. Each subtype is defined by the presence/absence of unique molecular markers and exhibits unique prognostic features [4,5,6,7]. The luminal subtypes, luminal A and luminal B, express the estrogen receptor (ER) and are deemed ER positive (ER+). They are more differentiated and more responsive to hormonal treatment, and as such are less aggressive and have more favorable prognosis. Whereas estrogen receptor negative (ER−) tumors, the HER2 overexpressing, the basal-like and the “claudin low” are more aggressive and have less favorable outcomes. Yet still a further subgroup of breast cancer is the triple negative breast cancers (TNBC), of which there are now at least six types with unique characteristics identified to date [8]. In addition to being ER−, these tumors are progesterone receptor negative (PR−) and HER2− as well.

### 1.1. Breast Cancer Progression and Metastasis

Metastasis of breast cancer to secondary sites in bone, liver, brain and lungs is largely incurable and is the principle cause of death in breast cancer patients [9,10,11,12]. It is a complex multistep process which involves the dissemination of cancer cells from the primary tumor mass to anatomically distant organs [13]. The metastatic cascade is a succession of cell-biological events that involve local invasion through the extracellular matrix and stroma cell layer, intravasation into the systemic circulation, survival during transportation through the vasculature, extravasation into distant tissues, establishment of micrometastasis in distant organs and finally colonization of these cells to form secondary tumors [14].

To achieve metastatic potential, a few cancer cells initially acquire the ability to evade and survive the homeostatic mechanisms of the organism. These cells are selected from the genetically heterogeneous lineages of cancer cells within the microenvironment of the tumor’s origin and continue to proliferate [15]. The continuous expansion of these rare tumor cells that express the genes necessary to initiate the metastatic cascade gives rise to clonal descendants that comprise the metastatic lesion [16,17].

### 1.2. Epithelial Mesenchymal Transition (EMT)

As epithelial cells convert from the non-invasive to the invasive phenotype, cells become anchorage independent and exhibit enhanced motility as well as increased aggressiveness. Epithelial mesenchymal transition (EMT) is thought to be a major mechanism for this conversion [16,18]. During EMT, epithelial cells acquire a mesenchymal-like phenotype via disruption of intercellular adhesion and resulting in enhanced motility (for review, [18,19,20]). Driving the EMT process are several transcription factors, such as Slug, Snail, Twist, Zeb1 and Zeb2 [20,21,22,23,24,25,26]. Up regulation of these transcription factors may cause dysfunctional cell-cell adhesion and loss of cell-cell junctions, thereby allowing the dissemination of tumor cells from the primary sites [19]. While the mechanism for the induction of EMT has clearly shown repression of the junctional adhesion molecules such as E-cadherin, definitive involvement of the tight junction (TJ) proteins has not been demonstrated until recently [19,21]. Emerging evidence now show that TJ proteins as well, may also be key players in the EMT process in many cancers including breast cancer [27,28,29].

## 2. Tight Junction (TJ) Proteins: The Claudins

**Figure 1:**
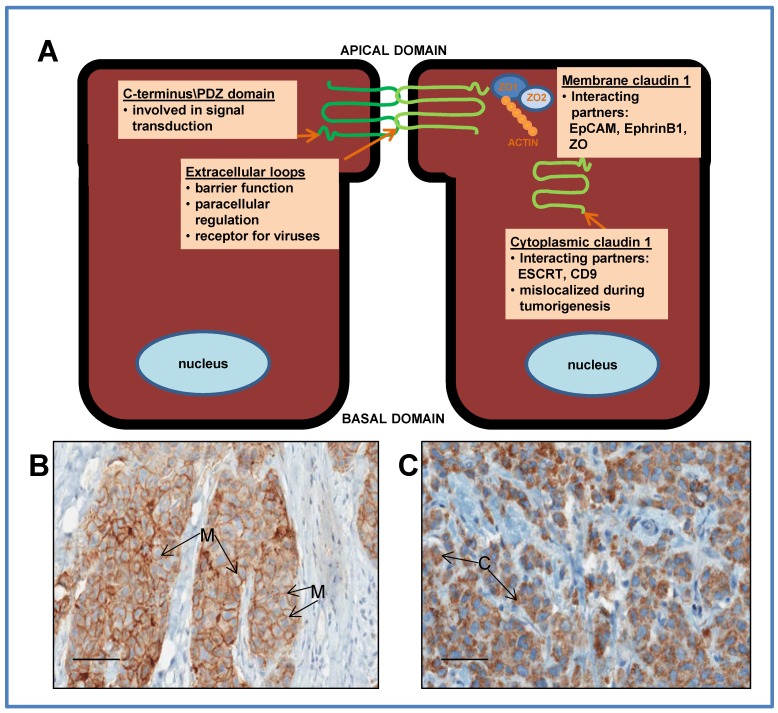
(**A**) Schematic representation of ductal epithelial cells, depicting the relative location and function of the claudin 1 protein in the membrane at the tight junction, and in the cytoplasm. The protein has 4 transmembrane domains with 2 extracellular loops with amino and carboxy terminal tails extending into the cytoplasm. The extracellular loops are essential for maintaining tight junction function, epithelial barrier integrity and regulating paracellular ion permeability. The *C*-terminus interacts with many signaling pathways through a PDZ domain. Through this domain, claudin 1 interacts with other tight junction proteins such as zona occludins (ZO). In addition to a tight junction function, membrane claudin 1 may act as a tumor facilitator by promoting collective migration during breast cancer progression; (**B**) Immunostaining of a breast tumor with claudin 1 antibody depicting membrane (M) staining and (**C**) depicting cytoplasmic (C) staining. Bars = 50 μm.

TJs form the closest contacts between adjacent cells along the apical border of the epithelial cell membrane [30]. They are comprised of a network of strands that encircle the cells, resulting in a barrier that separates apical and basolateral fluid compartments on opposite sides of the epithelial cell layer, thereby preventing paracellular movement of substances. This “paracellular seal” therefore, separates the extracellular fluid from the lumen (Figure 1). Additionally, TJs regulate the flow of nutrients and small ions between cells. They are also involved in maintaining the integrity and architecture of the cell and forms connections to the actin cytoskeleton [31,32]. A disruption of TJs during tumorigenesis generally leads to invasiveness, loss of cohesion, and lack of differentiation in cancer cells (for review, [33]).

The claudins are a family of integral membrane proteins (24 identified to date), central to the formation of TJs [34,35]. By regulating the passage of small ions and nutrients between cells, they maintain cell homeostasis and cell-cell communication [36]. Additionally, the claudins are critical for the maintenance of cellular polarity and are also involved in cellular growth and differentiation [37]. Several family members have been shown to be involved in cellular signaling [38,39]. Members of this family of proteins possess four transmembrane domains with two extracellular loops, with an amino and carboxyl terminal tail extending into the cytoplasm [39]. The extracellular loops are essential for maintaining TJ function and epithelial barrier integrity [40]. Moreover, the first extracellular loop of the claudin family has been identified as an important region of the protein for regulating paracellular ion permeability [41]. Variations in this first extracellular loop give rise to differences in the paracellular charge selectivity among members of the claudin family, whereas the *C*-terminus interacts with many signaling pathways and tight junction proteins through a PDZ domain. Through this domain, claudins interact with multiple TJ proteins [42,43]. The dysregulation of claudins in several cancers is now well documented (for review, [38,44,45,46,47,48,49]).

## 3. Claudin 1

Claudin 1, the first claudin family member identified, forms the backbone of the TJ strand [36,50]. Knockdown experiments of claudin 1 in mice have definitively demonstrated that it is essential for the epidermal barrier function [36]. The first extracellular loop possesses a key sequence motif capable of regulating TJ structure and function and is critical for epithelial barrier integrity [40]. As with all claudin family members, a PDZ binding domain is found within the intracellular *C*-terminal of the claudin 1 protein [39,51]. In claudin 1, this PDZ domain has been shown to interact with the zona occludins (ZO1 and ZO2), which connect to several signaling pathways. Interestingly, this domain in claudin 1 has been demonstrated to be important for and a target for different viral oncoproteins [52,53,54]. Further, the first extracellular loop of claudin 1 was shown to mediate uptake of the Hepatitis C Virus (HCV) [55], and more recently, the Dengue virus [56], thus identifying the claudin 1 protein as a receptor for viruses. Such a role has implications for claudin 1 as a potential target for antibody treatment of viral diseases, as well as in cancer therapy [57,58,59].

### 3.1. Dysregulation of Claudin 1 in Cancer

A dysregulation of claudin 1 in cancer has been well documented, and both a loss and gain has been associated with several cancers including oral, lung, prostate and gastric carcinoma as well as breast cancer [60,61,62,63,64,65]. In oral squamous cell carcinoma [44] and in breast cancer [61], the loss of claudin 1 has been associated with higher recurrence status and shorter disease free survival. In lung adenocarcinomas and hepatocellular carcinoma, enhanced invasiveness has also been demonstrated in association with claudin 1 loss [62,63]. In the latter, a down-regulation of claudin 1 has been associated with poor differentiation, portal invasion and low patient survival rates [63]. Conversely, the over expression of claudin 1 has been reported and was shown to increase cell invasion in colon [45,66,67], colorectal [68,69,70], oral squamous cell carcinoma [71] and melanoma [47]. Collectively, these studies provide evidence that claudin 1 can both promote or suppress tumorigenesis.

Interestingly, aberrant expression of claudin 1 has been observed not only in different cancers but in different histological subtypes of the same cancer. The over expression of claudin 1 in type II seropapillary endometroid carcinoma can distinguish it from type I which exhibits low claudin 1 expression [72].

### 3.2. A Putative Tumor Suppressor Role in Invasive Human Breast Cancer

Although not definitively proven, claudin 1 has long been postulated to be a tumor suppressor in breast cancer, as it was observed to be frequently down regulated or absent during disease progression. To lend further support to this hypothesis, a correlation between down regulation of claudin 1 and disease recurrence was also reported in a study involving 83 patients (57 patients with non-recurrent breast cancer and 26 patients with recurrent disease) [61]. More recently, in a much larger study using tissue microarray (TMA) strategies, Blanchard *et al.* [73], showed that only a small percentage of ER+ breast cancers were positive for claudin 1 whereas a significantly higher level of ER− tumors exhibited claudin 1 positivity. Further, *in vitro* studies showed that re-expression of claudin 1 alone was sufficient to induce apoptosis in a human breast cancer cell line, MDA-MB361 [74], and a loss of expression led to neoplastic transformation of mammary epithelial cells [75]. Claudin 1 has also been shown to be sufficient to exert TJ-mediated paracellular sealing in metastatic breast cancer cells in the absence of other TJ proteins [76].

There have been many speculations about the mechanisms responsible for claudin 1 loss in ER+ breast cancers. A search for mutations in the promoter or coding region of the claudin 1 gene has been futile as an explanation for the down regulation of claudin 1 in breast cancer [77]. Recent reports show that epigenetic factors, specifically transcriptional repression by methylation of the claudin 1 promoter near the CpG islands, may be partly responsible for claudin 1 down regulation [78,79]. As well, miRNA regulation may be another possible mechanism by which claudin 1 is deregulated in breast cancer. A down regulation of both claudin 1 mRNA and protein by miR-155 in ovarian cancer cells [80] was recently reported. Interestingly, miR-155 was found to be elevated in the blood of breast cancer patients and was associated with tumor progression [81,82]. It is therefore plausible that miR-155 may play a role in down regulating claudin 1 in breast cancer.

### 3.3. A Promoter of EMT, Cell Migration and Invasion

Claudin 1 involvement in EMT has also been well documented in several cancers [28,32]. Claudin 1 has been shown to have a causal role in EMT and malignant progression [32,45,66,83]. It has the ability to directly promote EMT through its interaction with defined EMT-related transcription factors and signaling pathways [23]. For example, in human liver cells, claudin 1 induced EMT through the activation of the transcription factors Slug and Zeb, which were regulated via the c-Abl/Raf/Ras/ERK signaling axis [32,84]. In colon cancer, modulation of β-catenin/Tcf signaling and E-cadherin expression and localization has been shown to play an important role in claudin 1-dependent regulation of EMT [85]. In breast cancer, a claudin-low subtype has been one of the more recent molecular subtypes identified. Claudin-low tumors characteristically exhibit low expression of claudins 1, 3, 4, and 7 [7,86], an up regulation of EMT markers and are enriched in stem-like cells [6,87]. In an earlier study, Sarrio *et al.* [88] suggested that in breast cancer, EMT likely occurs within a specific genetic context, the basal-like phenotype. In a large TMA study consisting of 479 patient biopsies, dysregulation of EMT markers, as well as an overexpression of proteins involved in extracellular matrix remodeling and invasion, was associated with the basal-like phenotype of breast cancers. In human breast cancer cell lines we have shown that either overexpression or inhibition of claudin 1 can alter the expression of EMT related molecules [27,89]. It has also been previously demonstrated that claudin 1 is a target for EMT markers, such as beta-catenin and Slug/Snail [90,91,92,93,94]. The latter, shown to bind to the E boxes in the claudin 1 promoter [23] and repress transcription. Recently, Hou *et al.* [95] further demonstrated that claudin 1 can also promote EMT *in vivo*. These authors showed that Stanniocalcin 2 (STC2) promoted breast cancer metastasis in mice through its interaction with claudin 1, which directly led to an alteration of the expression of the EMT molecules including Zeb1, Slug, and Twist [95].

### 3.4. Interaction with the Extracellular Matrix

The matrix metalloproteinases (MMPs) are a family of zinc-dependent endopeptidases which can degrade and remodel the extracellular matrix (ECM) and its constitutive proteins, during both normal processes (such as morphogenesis and tissue repair), and pathological processes (such as the facilitation of cell migration and invasion) during EMT [96]. However, in the normal intestinal epithelium, independent of TJs, claudin 1 and 7 can form complexes with α2 integrin to regulate cell-matrix interactions and increase the expression of MMPs [97]. Claudin 1 interaction with the MMPs in the extracellular matrix also demonstrates its involvement in invasion and tumor progression. In cancer, claudin 1 increased cell motility and invasiveness through its deregulation of MMPs such as MMP1, MMP-2 and MMP-9 directly [71,98].

In breast cancer claudin 1 co-localized and directly interacted with membrane type MMP-1 and pro-MMP-2 and mediated its activation [98]. MMP-2 cleaved ECM proteins such as collagen I and collagen IV [96], as well as laminin-5 [71], by binding to integrin, an ECM receptor to facilitate cell invasion. This activation of MMPs by claudin 1 does not appear to be related to its TJ functions as the co-localization of claudin 1 with membrane type MMP-1 was not found at TJ strands but instead was located diffusely in the membrane and primarily in the cytoplasm [98].

This suggests a dual role for claudin 1 in its interactions with the ECM. Claudin 1 may form complexes with ECM proteins to stabilize cell-matrix interactions, and/or assist in the degradation of the ECM through MMPs during cell invasion.

## 4. New Insights into the Role of Claudin 1 in Breast Cancer

### 4.1. A Leading Role in Collective Migration

Both *in vitro* and *in vivo* studies reveal an emerging role for claudin 1 in breast cancer. The concept that there are two main patterns by which cancer cells migrate, collective migration or single cell migration, has been described several years ago, but the mechanisms are not well understood. Single cell migration, as the name implies, refers to the detachment of single epithelial cells from the tumor, and, facilitated by the acquisition of mesenchymal characteristics, then proceed to navigate through the ECM to metastasize to distal sites [11,99]. Collective cell migration, however, is characterized by the migration of whole groups of cells interconnected by adhesion molecules and other communication junction proteins [100]. This collection of cells then detaches from the tumor mass and penetrates into the surrounding tissues. During collective migration, the “group” is observed to have a “leading front” and utilizes integrins and proteases to facilitate migration. Strikingly, clear differences in gene expression exist between “leader” cells and “followers” [101,102,103]. Collective migration has now been demonstrated in several cancers, including breast cancer, and can occur through partial EMT mechanisms or can occur totally independently of EMT [102,104].

Claudin 1 plays a key role in facilitating the collective migration of tumor cells. Work by Giampieri *et al.* [102] showed the involvement of claudin 1 in migration was through partial EMT mechanisms. In rat mammary carcinoma, cancer cells displayed single cell migration under high transient expression of TGFβ, leading to blood-borne metastasis. However, following the inhibition of TGFβ expression, the cancer cells moved collectively and lead to lymphatic invasion. Importantly, it was also demonstrated that the expression of TGFβ was tightly regulated by claudin 1, such that, when claudin 1 levels were high, TGFβ expression was inhibited, leading to collective migration. More recently, Fortier *et al.* [105], showed that claudin 1 had the potential to induce collective migration, independent of EMT pathways. Using cervical carcinoma cells, they showed that knockdown of keratin (K8/18), a marker of epithelial cells, increased the collective migration and invasiveness of the cancer cells without modulating EMT markers. Once again, they identified claudin 1 as a major player in this process. They also observed that sub-confluent sheets of cells along the leading edge exhibited intense cytoplasmic and nuclear staining for claudin 1, while “follower cells” demonstrated a continuous membrane-staining pattern. As well, claudin 1 simultaneously exhibited high levels of expression in some cells while low levels of expression were observed in neighboring cells, depending on their position in the “pack”. Based on such positioning, it was proposed that cytoplasmic claudin 1 plays a role in cell motility, while junctional claudin 1 facilitates increased cell cohesion and consequently, and in so doing, facilitated collective cell migration.

### 4.2. More Than a Tumor Suppressor? “Claudin High” Breast Cancers

**Figure 2:**
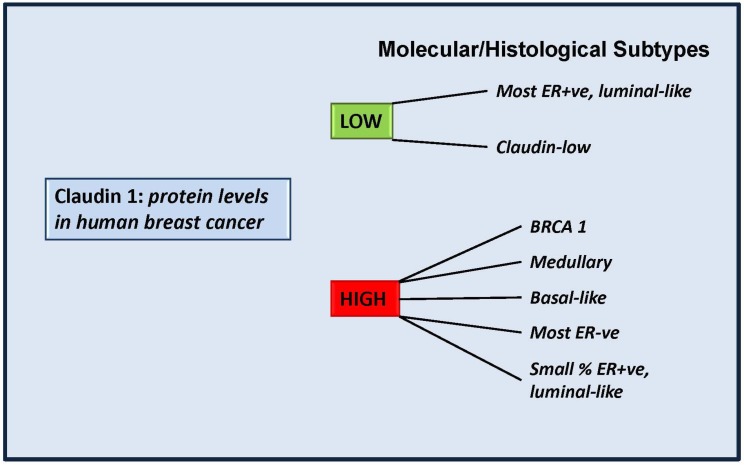
Claudin 1 levels in molecular/histological subtypes of breast cancer. Some molecular subtypes of human invasive breast cancer have been shown to exhibit low levels of claudin 1, in line with studies which suggest claudin 1 plays a tumor suppressor role in those tumors. However some aggressive forms exhibit high endogenous levels of claudin 1 and it is still to be determined whether in these tumors, claudin 1 is playing a tumor-facilitating role. BRCA1: a hereditary breast cancer. Patients carry a mutation of the breast cancer 1 (BRCA1) gene. Medullary: a subset of basal-like carcinomas that exhibit a high rate of p53 mutations [108].

Despite the fact that many laboratories, including our own, suggest that claudin 1 is a tumor suppressor in invasive breast cancer, our studies also suggest that this is not the case for all breast cancers [83]. We have additionally shown that ER− tumors exhibited a significantly higher frequency of positivity for claudin 1, while ER+ tumors often exhibit very diminished or complete loss of claudin 1 expression [73]. Furthermore, we identified a significantly high association with claudin 1 and the basal-like subtype of breast cancers [73], an aggressive form of breast cancer, which to date remains poorly characterized [106]. In support of these observations, Lu *et al.* [86], also recently showed high levels of claudin 1 in the basal-like subtype. These authors also identified high claudin 1 levels in a small percentage of luminal and HER2 subtypes, further highlighting the heterogeneous nature of this disease and the need to better characterize molecular subtypes already identified. Moreover, we also showed that there was a positive relationship between high claudin 1 protein levels in the tumor and patient age [27]. However, we were unable to identify any significant association between claudin 1 levels and patient survival. Interestingly, increased levels of claudin 1 have also been reported for medullary and BRCA1-type breast cancers as well, and it was further proposed that claudin 1 expression can be used to discriminate mutation carriers from sporadic breast cancer cases [107], (Figure 2).

Indeed it could be speculated that the high levels of claudin 1 observed in breast cancer may be attributed to a “bystander effect” and do not contribute or participate in tumorigenesis. However a direct role for claudin 1 in altering the migration and proliferation rates of several human breast cancer cell lines, has now been demonstrated [27,89]. In particular, we showed that the knockdown of claudin 1 in a basal-like subtype human breast cancer cell line, resulted in reduced cancer cell migration [27]. Thus, the high levels of claudin 1 expression in some basal-like breast cancers has led to further speculations that claudin 1 may be a tumor-facilitator in this breast cancer subtype [83], as has been shown for claudin 1 in melanomas, colon cancer, and oral squamous cell carcinomas [45,47,66,67,71]. Further support for such speculations comes from studies by Suh *et al.* [32], who recently demonstrated that in hepatic carcinoma cells claudin 1 directly participated in the molecular signaling that contributes to tumor malignancy. This may also be the case in breast cancer.

### 4.3. Cytoplasmic Mislocalization in Breast Cancer

The appropriate subcellular localization of proteins is essential in providing a physiological context to their function. However, subcellular mislocalization of membrane proteins can often occur at the onset and progression of various human cancers. The mislocalization of claudin 1 to the cytoplasm, has been observed in several cancers [27,28,45,47,73,109,110]. For example, in colon, oral squamous cancer and melanomas, the subcellular localization of claudin 1 in cytoplasm has been associated with tumor progression [45,47,66,67,71]. Cytoplasmic localization was found to enhance tumor progression and the metastatic potential of the cancer cells. Interestingly, in melanoma, when claudin 1 was found in the nucleus it appeared to have no impact on invasion and metastasis [47].

It was hypothesized that the PDZ-binding motif within the *C*-terminal was the structural component of claudins integral to their correct localization. However, this was shown not to be the case as it was demonstrated that claudin 1 mutants bearing a mutated PDZ binding domain, were still incorporated into apical TJs [111]. However, the removal of the entire *C*-terminal tail resulted in its mislocalization to the cytoplasm, suggesting that it was the cytoplasmic juxta membrane region, not the PDZ-binding domain, that was structurally essential for claudin 1 localization to the TJ [111]. Moreover, it was determined that the carboxyl-terminal region contained numerous phosphorylation sites that may contribute to that process [28]. Mimicking constitutive phosphorylation at Protein Kinase A (PKA)/Protein Kinase C (PKC) sites, was shown to cause cytoplasmic claudin 1 localization [112].

### 4.4. Interaction with Unique Subcellular Partners

Recently, a number of claudin 1 interacting proteins have been identified that may play a direct role in the amount, function and subcellular localization of claudin 1 in breast cancer (Table 1).

**Table 1:** Putative interacting partners of claudin 1 in breast cancer.

Protein	Description	Location	References
Ephrin B1	A transmembrane protein involved in intrinsic cell signaling	Membrane	[113,114]
ESCRT	Required for proper protein transport and maintenance of epithelial cell polarity	Cytoplasm	[115]
CD9	A transmembrane protein that plays a role in cell fusion and invasion	Membrane	[116]
EpCAM	A transmembrane surface glycoprotein	Membrane	[117]

**Ephrins:** The Ephrin (Eph) family receptors (protein tyrosine kinases), and its ligands can induce bidirectional signaling in epithelial cells via cell-cell contact [118]. Claudin 1 has been shown to have a unique relationship with Ephrin B1 [114], a transmembrane protein involved in intrinsic cell signaling [118]. The extracellular domains of claudin 1 have been shown to facilitate the phosphorylation of Ephrin B1 interaction through binding of both domains to Ephrin B1 resulting in cell-cell contact. This interaction with claudin 1 mediates the tyrosine phosphorylation of Ephrin B1 in its cytoplasmic region in a manner dependent on claudin 1. The phosphorylation of Ephrin B1 further mediates cell migration and invasion through the downstream exocytosis of matrix metalloproteinases ultimately leading to changes in levels of cell-cell adhesion [118]. Interestingly, interrogation of the claudin 1 amino acid sequence has revealed conserved Eph phosphorylation sites [113], which indicates that the claudins in turn may also be phosphorylated by members of the Eph family, revealing that the relationship between claudin 1 and the Ephrin family may not be as straightforward as it first appears. Moreover, it has been observed that Eph receptors are frequently overexpressed in cancerous tissues [119]. Understanding the interactions of the Eph family, their ligands and claudin 1 will be an important novel area of study with regards to breast cancer progression.

**The ESCRT machinery:** It has been shown that the intracellular accumulation of claudin 1 can occur as a result of defective interactions with the Endosomal Sorting Complex Required for Transport (ESCRT) machinery, a collection of cytosolic proteins required for proper protein transport and maintenance of epithelial cell polarity [120]. ESCRT aids in maintaining cell polarity by regulating the recycling and trafficking of membrane-bound proteins, including claudin 1, via endocytic mechanisms [115]. Thus, ESCRT plays an important role in claudin 1 transport to the membrane. Inhibition of ESCRT function causes claudin 1 to accumulate in the cytoplasm. This leads to weakening and disassembly of TJs and loss of cellular polarity. In epithelial cancers, including hepatocellular carcinoma and pancreatic tumors, components of ESCRT show reduced expression [115]. Moreover, loss of ESCRT function in cancer cells has been associated with increased proliferation, together with the acquisition of a less stable tissue architecture [115], two of the key features acquired by tumor cells [121]. Such observations suggest that ESCRT may facilitate the tumor-suppressive role of claudin 1 in breast cancer.

**CD9:** The tetraspanin CD9 has recently been identified as an interacting partner of claudin 1. Claudin 1 is one of a few known interacting partners of CD9. Tetraspanins are transmembrane proteins that typically reside on cell surface and do not generally function as ligands or receptors [116]. Rather, they assemble themselves with other proteins to form microdomains [122,123,124,125]. Tetraspanins act to regulate cell fusion, invasion, migration and differentiation [126].

In breast cancer, the interaction of claudin 1 with CD9 has been shown to coincide with its subcellular co-localization in breast cancer. This has now been demonstrated in multiple cell lines, including the human breast cancer cell lines MDA-MB231 (basal-like subtype) and MCF7 (luminal A subtype) [116]. Interestingly, CD9-claudin-complexes do not appear to reside in TJs, but rather in the cytoplasm and may be indicative of a role for claudin 1 other than that of a TJ protein. CD9 was shown to have a destabilizing effect on claudin 1. Depletion of CD9 diminished the half-life of non-junctional claudin 1 by approximately 50% [116]. It has also been suggested that CD9 prevents claudin 1 from associating with the TJ [116]. This could indeed reflect a normal regulating process, or is a contributing factor to the increased EMT and consequently migration that results from TJ disruption. If indeed CD9 prevents claudin 1 from associating with the TJ, then this could promote tumor progression. In support of such a hypothesis, it has been shown that the over expression of CD9 in human breast cancer promotes the development of bone metastases [127].

**EpCAM:** Epithelial cell adhesion molecule (EpCAM), also known as CD326, is a transmembrane surface glycoprotein found expressed in epithelia and some invasive carcinomas [128]. EpCAM is generally localized to the lateral interfaces of polarized epithelial cells, but does not co-localize with TJs [117]. EpCAM exhibits pro-oncogenic activity, by promoting cell proliferation and motility and metastasis [129]. As a result, the therapeutic potential of EpCAM antibodies is being actively explored.

EpCAM has recently been shown to play an important role in regulating the composition and function of TJs through specific interaction with claudin 1. Wu *et al.* [117] recently demonstrated a physical interaction between claudin 1 and EpCAM in epithelial cells. They showed that this relationship protected claudin 1, which is continually trafficked from the membrane to the lysosome, from degradation and regulates its amount and function in the cell. Moreover, this physical contact was shown as necessary for the stabilization of claudin 1. The importance of such interfacing was further highlighted by experiments, which demonstrated that knocking down EpCAM in cancer cells resulted in increased overall TJs in the membrane and diminished intercellular claudin 1. An interaction between EpCAM and claudin 1 has not yet been demonstrated in breast cancer but a similar interaction may account for one of the mechanisms by which high levels of claudin 1 accumulate in the cytoplasm of some aggressive breast cancer cell lines and tumors, and warrants further investigation.

## 5. Future Perspectives

The void in our knowledge about the ever-emerging breast cancer molecular subtypes presents an insurmountable challenge to finding effective strategies for managing this disease. In the healthy mammary gland, TJ permeability can be reproduced readily in response to a number of physiological stimuli [130], and thus tight regulation of cell permeability is critical for maintaining homeostasis. Enhancing our understanding of the regulation of TJ permeability, or lack of it thereof, in pathological states is critical. Indeed, research into the development of approaches to modulate barrier function for efficient drug delivery continues to receive much attention [131], as dysregulation of the claudin family of TJ proteins leads to tumor progression [33,132].

The loss of cell-cell adhesion is a crucial step in EMT, and thus strategies to overcome the altered expression of TJ proteins in cancerous tissues are attractive as they could eventually lead to the development of effective therapeutic management for treating and possibly preventing human cancers.

Claudin 1 is of particular interest in the quest to identify new targets for breast cancer therapy as, (1) increasingly it has been shown to have direct involvement in the migration and proliferation rates in several cancers, including breast [44,45,47,112] and (2), it is a receptor, and thus, a port of entry for viruses [55,133]. Recently the HCV gene E2 was shown to be mainly involved in viral entry into the host via claudin 1 [134]. Such knowledge of this interaction between host cell surface receptors and viral factors may offer new therapeutic options in patients with aggressive tumors that exhibit high claudin 1 levels. Furthermore, it has been suggested that the identification of a critical claudin 1 extracellular loop motif (claudin-1 53–80) [40], capable of regulating TJ structure and function, may offer a useful adjunct to treatment that require drug delivery across the epithelial barrier.

As well, the pattern of expression of claudin 1 in different molecular subtypes indicates that claudin 1 may be a useful biomarker for the detection, diagnosis and treatment of breast cancer and may also serve as a tool for predicting disease progression. [27,73]. It has recently been shown that when claudin 1 was used in conjunction with four other markers, this cohort of markers was a useful predictive indicator for breast cancer patients [135], suggesting it may have utility as a potential biomarker for breast cancer. Decreased claudin 1 expression is positively correlated with frequency of recurrence and shorter disease-free intervals in breast cancer [61]. High claudin 1 levels have also been shown to be associated with some aggressive forms of breast cancers, including inflammatory breast cancers, hereditary breast cancer, some high grade invasive ductal carcinoma [86] and the basal-like subtypes. Collectively, these observations suggest the potential value of claudin 1 in identifying specific groups of breast cancer patients and present new opportunities for developing effective therapeutic strategies for managing breast cancer.
